# The Incorporation of Immunotherapy and Targeted Therapy Into Chemoradiation for Cervical Cancer: A Focused Review

**DOI:** 10.3389/fonc.2021.663749

**Published:** 2021-05-26

**Authors:** Otasowie Odiase, Lindsay Noah-Vermillion, Brittany A. Simone, Paul D. Aridgides

**Affiliations:** Department of Radiation Oncology, SUNY Upstate Medical University, Syracuse, NY, United States

**Keywords:** cervical cancer, radiotherapy, chemotherapy, immunotherapy, angiogenesis inhibitors

## Abstract

In 2011 the Food and Drug Administration (FDA) approved anti-vascular endothelial growth factor (VEGF) therapy, bevacizumab, for intractable melanoma. Within the year, immunotherapy modulators inhibiting cytotoxic T-lymphocyte-associated protein 4 (CTLA-4) and programmed cell death protein 1 (PD-1) were approved in addition to programmed death-ligand 1 (PD-L1) antibodies in 2012. Since then, research showing the effectiveness of targeted therapies in a wide range of solid tumors has prompted studies incorporating their inclusion as part of upfront management as well as refractory or relapsed disease. For treatment of cervical cancer, which arises from known virus-driven oncogenic pathways, the incorporation of targeted therapy is a particularly attractive prospect. The current standard of care for locally advanced cervical cancer includes concurrent platinum-based chemotherapy with radiation therapy (CRT) including external beam radiation therapy (EBRT) and brachytherapy. Building upon encouraging results from trials testing bevacizumab or immunotherapy in recurrent cervical cancer, these agents have begun to be incorporated into upfront CRT strategies for prospective study. This article will review background data establishing efficacy of angiogenesis inhibitors and immunotherapy in the treatment of cervical cancer as well as results of prospective studies combining targeted therapies with standard CRT with the aim of improving outcomes. In addition, the role of immunotherapy and radiation on the tumor microenvironment (TME) will be discussed.

## Introduction

Treatment options for early-stage cervical cancer include surgery or primary radiation with or without chemotherapy (1). Surgery in the form of radical hysterectomy is indicated for non-bulky and early stage disease although definitive radiotherapy has similar efficacy. For patients with IB-IIA disease, a randomized trial of 343 women compared surgery versus radiation with initial results showing five-year overall survival (OS) of 83% in both groups (2). Rates of severe morbidity were higher (p = 0.0004) in those receiving surgery upfront (28%) compared to radiotherapy (12%), which was attributed to increased use of combination surgery and adjuvant radiation in the surgery arm. Long-term follow up continued to show similar twenty-year OS rates of 72% with surgery and 77% with primary radiotherapy (p = 0.280) (3). Multivariate analysis identified large tumor size (p = 0.008), adenocarcinoma histology (p = 0.020), and positive lymph node status (p <0.001) as negative risk factors.

For bulky or locally advanced stage disease, the addition of cytotoxic chemotherapy to radiation has been the subject of extensive study. The seminal Gynecological Oncology Group (GOG) 120 trial examined 526 women with untreated stage IIB, III, or IVA cervical cancer. Patients received EBRT with random assignment to one of three concurrent CRT regimens: cisplatin, cisplatin plus 5-fluorouracil, or oral hydroxyurea. Patients receiving either cisplatin-containing arm had improved rates of OS and progression free survival (PFS) (4). In a similar cohort to GOG 120, Radiation Therapy Oncology Group (RTOG) 90-01 examined 403 women with stages IIB–IVA, stages IB to IIA with bulky tumors, or positive pelvic lymph nodes. This randomized study compared extended field radiotherapy (EFRT) alone to CRT consisting of pelvic radiotherapy with concomitant fluorouracil and cisplatin. The 90-01 results met early release criteria due to CRT garnering a significant OS and disease-free survival (DFS) benefit compared to EFRT alone. Long-term follow-up confirmed significantly improved eight-year OS of 67% with CRT compared to 41% with EFRT (p <0.0001) (5). RTOG 90-01 was the tipping point of a culmination of studies that caused a dramatic change in National Institutes of Health recommendations to concurrent CRT as the standard of care for cervical cancer, most notably for stage IB3–IVA disease (4–7). The focus of this review will be to examine studies that are completed or in development combining newer therapeutic agents, including angiogenesis inhibitors and immunotherapy, with CRT in the management of cervical cancer.

## Angiogenesis Inhibition

### Efficacy of VEGF Inhibitors in Cervical Cancer

There is evidence that VEGF plays a role in human papilloma virus (HPV) mediated oncogenesis of cervical cancer, including through activity of oncoprotein E5 to upregulate the VEGF angiogenesis pathway (1). VEGF is a growth factor responsible for the proliferation, migration, and survival of endothelial cells. Increased levels of VEGF have been associated with advanced stages of cervical cancer, as well as worse PFS and OS (8–10). Bevacizumab is an anti-VEGF monoclonal antibody that binds to VEGF proteins expressed on tumor cells (11, 12). The GOG 227C study evaluated the use of bevacizumab in 46 patients with recurrent cervical cancer (**Table 1**). This Phase II study showed that bevacizumab as monotherapy was tolerable and improved PFS and OS as a second, or third line treatment when compared to historical GOG study controls (13). Few grade 3 or 4 adverse events were reported as well as one grade 5 infection.

**Table 1:** Clinical trials using anti-vascular endothelial growth factor (anti-VEGF) in cervical cancer with prior or concurrent treatment with chemoradiation.

Study	Phase Study Population Subject number (n)	Treatment	Results
**GOG 227C** Bevacizumab in the Treatment of Persistent or Recurrent Squamous Cell Carcinoma of the Cervix (13)	Phase II Recurrent, 83% had prior radiation, all had prior chemotherapy n = 46	Bevacizumab every 3 weeks until disease progression or prohibitive toxicity	Median PFS: 3.40 months (95% CI, 2.53 to 4.53 months) OS: 7.29 months (95% CI, 6.11 to 10.41 months) Adverse Events: grade 3 or 4 Hypertension (n = 7) Thrombo-embolism (n = 5) Gastro-intestinal (n = 4) Grade 5 infection (n = 1)
**GOG 240** Incorporation of Bevacizumab in the Treatment of Recurrent and Metastatic Cervical Cancer (14)	Phase III Recurrent, persistent, or metastatic, 75% had prior concurrent cisplatin-radiation n = 452	2 × 2 design First randomization: cisplatin + paclitaxel or topotecan + paclitaxel Second randomization: with or without bevacizumab every 3 weeks	Median OS: 16.8 months in chemotherapy + bevacizumab versus 13.3 months in chemotherapy alone (HR 0.77;95% CI 0.62–0.95; p = 0.0068
**RTOG 0417** Efficacy of Bevacizumab in combination with definitive radiation therapy and cisplatin chemotherapy in untreated patients with locally advanced cervical carcinoma (15)	Phase II Newly diagnosed with bulky/locally advanced stage IB-IIIB n = 49	Bevacizumab every 2 weeks × three cycles concurrent with cisplatin/pelvic radiation then followed by brachytherapy	Results at 3 years OS: 81.3% (95% CI, 67.2–89.8%) LF: 23.2% (95% CI, 11–35.4%) PAF: 8.4% (95% CI, 0.4–16.3%) DFS: 68.7% (95% CI, 53.5–79.8%)

GOG, Gynecologic Oncology Group; PFS, progression free survival; CI, confidence interval; OS, overall survival; HR, hazard ratio; LF, locoregional failure; PAF, para-aortic failure; DFS, disease free survival.

Building on these results, GOG 240 was a 2 × 2 phase III randomized trial of the addition of bevacizumab to two different chemotherapy regimens, cisplatin vs paclitaxel-topotecan. The majority (75%) of the 452 patients with recurrent, persistent, or metastatic cervical cancer (**Table 1**) had previously received cisplatin-based CRT. This study showed the addition of bevacizumab to chemotherapy was found to improve median OS from 13.3 to 17.0 months (hazard ratio 0.71 (98% confidence interval (CI), 0.54–0.95; p = 0.004) (14). In a subset of patients who had not received previous radiation, median OS was 24.5 months with bevacizumab added to chemotherapy versus 16.8 months in chemotherapy alone. Bevacizumab was associated with increased risk of grade 2 or higher hypertension (25% versus 2%), although no patients discontinued bevacizumab because of hypertension. In addition, thromboembolic events (grade 3 or higher) were higher with bevacizumab (8% *versus* 1%). Of particular importance is the risk of fistula (grade 3 or higher) with bevacizumab at 6% compared to <1% with chemotherapy alone, and all fistulas occurred in previously radiated patients. Fistula is a consistently reported rare toxicity of CRT regimens with brachytherapy with significant negative effects on quality of life (QOL). In GOG 240 there were no fistula associated surgical emergencies, instances of sepsis or death and although there was a reported decrease in QOL measures in bevacizumab receiving groups, this was non-significant (16). The toxicity profile of bevacizumab for these reasons in the refractory or metastatic setting therefore merits individualized and careful consideration (16, 17). The median post-disease progression-OS was not reduced in the bevacizumab vs chemotherapy-alone group at 8.4 *vs* 7.1 months respectively, lending support to addition of bevacizumab as part of upfront treatment in this setting rather than following next progression. Overall the GOG 240 study results prompted FDA approval in 2014 and established a standard of care for patients with metastatic or recurrent cervical cancer for the addition of bevacizumab to systemic chemotherapy (1).

### Anti-VEGF and Radiation Therapy

While prior radiation was common for patients on GOG 227C (82.6%) and GOG 240 (75% received cisplatin CRT), the unknown effectiveness and toxicity profile of bevacizumab in combination with definitive CRT prompted prospective study on RTOG 0417 (**Table 1**) (13–15). RTOG 0417 was a phase II study combining bevacizumab and CRT in patients with untreated locally advanced cervical carcinoma (15). Unlike GOG 227C, RTOG 0417 was powered to specifically evaluate for toxicity as the primary endpoint. Secondary endpoints included OS, DFS, locoregional failure (LRF) as well as nodal failure associated with radiation and immunotherapy. The study specified the use of 40 mg/m^2^ weekly cisplatin and standard definitive pelvic radiation therapy with four field high energy photons totaling 45 Gray (Gy) in 25 fractions, 5 days per week to include external iliac lymph nodes. Intensity Modulated Radiation therapy (IMRT) was not permitted. Bevacizumab was given at 10 mg/kg every 2 weeks for three cycles during CRT. Brachytherapy followed at a dose of 40 Gy in one to two low dose rate treatments or 30 Gy in high high dose rate treatments with bevacizumab administered once during brachytherapy course. No maintenance bevacizumab was given. Two of the 46 patients developed grade 3 gastrointestinal (GI) adverse events, with no grade 4 or 5 events. Notably there were no GI fistulas or perforations reported. Hematologic toxicity was the most reported adverse event (nine grade 3, three grade 4). This study showed that the addition of bevacizumab to standard CRT for locally advanced cervical cancer was feasible and safe with respect to protocol-specified treatment related serious adverse events and adverse events. Initial outcomes were encouraging, as incorporation of bevacizumab with CRT resulted in 3-year OS of 81.3%, DFS 68.7% and LRF was 23.2%. An interesting but yet unstudied hypothesis would be to test the efficacy of adjuvant/maintenance bevacizumab following definitive management of locally advanced cervical cancer with CRT, given that in GOG 240 in the recurrent/metastatic setting bevacizumab combined with chemotherapy yielded response rates of 47% (18). We are not aware of an upcoming randomized trial in development evaluating bevacizumab with CRT for cervical cancer in the upfront setting. Possible reasons include toxicity concerns of bevacizumab including risk of fistula as reported in GOG 240, which all cases of fistula were in patients who had previous CRT. However, there were no fistulas reported on RTOG 0417. Also, the OS, DFS, and LRF outcomes on RTOG 0417 were fairly comparable to the CRT arm of RTOG 90-01. Within the NRG cooperative group, the addition of a Ribonucleotide Reductase Inhibitor (Triapine) was selected for randomized study (NRG GY006) given in addition to standard of care CRT for locally advanced cervical and vaginal cancer (19).

## Immunotherapy

### PD-L1 Inhibitors for Cervical Cancer Treatment

A majority (>95%) of cervical cancers originate from HPV, an overt carcinogenic factor in cervical cancer development. An increase in PD-L1 expression has been observed in HPV-related head and neck squamous cell carcinoma (SCC) (20). This is likely owing to the upregulation of PD-L1 expression in tumor cells by the E5, E6 and E7 oncoproteins (21). While PD-L1 expression is rare in normal cervical tissue, it is present in about 50% of cervical cancer T-cells, with several studies identifying PD-L1 as a strong prognostic factor as well as a treatment target for cervical cancer (20, 22, 23). Upregulation of PD-L1 on tumor cells leads to increased binding and inhibition of the PD-1 receptor on T-cells. This interaction allows tolerance of tumor antigens presented by major histocompatibility complex molecules and thus turns off the anti-tumor immune response. In addition to deactivation of cytotoxic T-cells, upregulation of PD-L1 causes release of tumor permissive T-helper cell type-2 cytokines in the TME. Blockade of this interaction is a potential treatment strategy that reverses the brakes that upregulation of PD-L1 puts on the immune response.

Pembrolizumab is a highly selective, fully humanized monoclonal antibody that binds to PD-1 and inhibits the PD-L1 pathway. The KEYNOTE-028 study was a phase Ib trial exploring the effects of pembrolizumab in advanced previously treated PD-L1 positive cervical cancer (24). This single arm trial included 24 patients with advanced cervical cancer whose disease failed to respond to prior systemic therapy and whose tumor or stromal tissue had PD-L1 expression of ≥1%. Most patients (62.5%) had received ≥2 previous lines of therapy. Patients received pembrolizumab at 10 mg/kg every 2 weeks for up to 24 months. Pembrolizumab monotherapy had an overall response rate (ORR) of 12.5% at a median follow-up time of 48.9 weeks, as well as no grade 4 adverse events or deaths. In the subsequent phase II study, KEYNOTE-158, patients with advanced cervical cancer were treated with pembrolizumab at 200 mg every 3 weeks, regardless of PD-L1 status (25). The ORR by Response Evaluation Criteria in Solid Tumours (RECIST), (version 1.1), was 12.2% with 10.2 months of follow-up. For patients with longer follow-up (at least 27 weeks) ORR increased to 27%. The results of KEYNOTE-158 prompted FDA accelerated approval of pembrolizumab in the second line treatment of advanced PD-L1 positive cervical cancer (20). It should be noted that many subsequent immunotherapy trials now utilize the immunotherapy-RECIST (iRECIST) criteria for evaluating response to therapy (26).

Nivolumab is another monoclonal antibody with a high affinity to PD-1. It blocks interaction of PD-1 on T-cells with PD-L1 and programmed death ligand-2 (PD-L2) on tumor cells and allows for tumor antigen-specific T-cell proliferation and cytokine release (27). The CheckMate-358 trial is an ongoing open-label, multicohort, phase I/II study of nivolumab in patients with virus-associated tumors including recurrent or metastatic cervical, vaginal, and vulvar cancers. Patients received nivolumab at 240 mg every two weeks until progression of disease or unacceptable adverse events. Of the 24 patients treated, 19 had cervical cancer. ORR in the phase I cohort was 26.3% for patients with cervical cancer, with a median follow-up of 31 weeks. In all 24 patients, the disease control rate (ORR + stable disease) was 70.8% (28).

### Combination PD-L1 Inhibition and CTLA-4 Inhibition

While PD-1 inhibition has shown promise in cancer therapy, combinatorial approaches that target both PD-1 and CTLA-4 pathways have also been employed. The combination of ipilimumab, a CTLA-4 inhibitor, and nivolumab has shown efficacy and is FDA approved for the treatment of melanoma (29). However, it is not well known how one agent may affect expression of the target for another agent. PD-L1 levels have been evaluated in tumors treated with ipilimumab in metastatic or recurrent cervical cancer patients who had progression after at least one line of platinum chemotherapy with pelvic radiotherapy (30). Thirteen of the 42 total patients had adenocarcinoma versus squamous cell carcinoma and 37/42 were known HPV positive. This study showed that PD-L1 expression at baseline and post immunotherapy did not increase significantly with treatment and was not an indicator of outcome. Median PFS and OS were 2.5 months (95% CI, 2.1–3.2 months) and 8.5 months (95% CI, 3.6 not reached; one patient was still alive) respectively. This study did show evidence of PD-L1 changes with CTLA-4 inhibitor monotherapy in patients with metastatic or recurrent cervical cancer post CRT.

### Combination PD-L1 or PD-1 Inhibition With Radiotherapy

Cemiplimab, a hinge-stabilized immunoglobulin-4 monoclonal antibody to the PD-1 receptor, exhibits a safety profile comparable to other anti PD-L1 agents. During its first in human study of 60 patients with solid tumors deemed to have no standard alternative therapeutic options, nine patients had either partial (7) or complete (2) responses to cemiplimab given concurrently to hypofractionated radiation (31). There were three cervical cancer patients treated, including one of the two patients in the study achieving complete response.

There are several cervical-cancer specific, ongoing or newly completed clinical trials exploring the new realm of adding immunotherapy to CRT concurrently, sequentially or both. NRG GY017 is a multi-faceted Phase I clinical trial studying immune activation differences in the timing of anti PD-L1, atezolizumab (**Table 2**) (33). This two-arm study has one arm receiving an upfront single dose of atezolizumab then continues with two treatments of atezolizumab concurrently with extended field CRT and image guided brachytherapy. The second arm receives three doses of atezolizumab concurrently with extended field CRT and image guided brachytherapy. IMRT will be used for its potential reduction in adverse events and regional node recurrence (39). Post-treatment positron emission tomography and computed tomography (PET-CT) scans, an often-utilized post treatment surveillance tool, will also be followed prospectively. Immune expression differences between the arms will be measured *via* clonal expansion of T-cell receptor beta in peripheral blood at baseline and on day 21 of treatment. It is hypothesized that immune responses of increased clonal numbers and in specific tumor associated clones will be shown in the treatment arm with the best clinical outcomes. Baseline and treatment PD-L1 expression in both arms will also be analyzed for outcome predictive value.

**Table 2:** Early results and ongoing clinical trials of immunotherapy with chemoradiation in cervical cancer.

Study	Phase Study Population Subject number (n)	Treatment	Results or Primary/Secondary Outcomes
**GOG 9929** Phase I study of sequential Ipilimumab in the definitive treatment of node positive cervical cancer (32)	Phase I Node positive cervical cancer n =34 (19 patients evaluated for endpoints)	Definitive Cisplatin + EFRT followed by Ipilimumab (CTLA-4 inhibitor)	Results 1 year: Ipilimumab Maximum Tolerated Dose: 10 mg/kg Disease Free Survival: 74%
**NRG GY017** Anti-PD-L1 (Azetolizumab) as an Immune Primer and Concurrently with EFRT for Node Positive Locally Advanced Cervical Cancer (33) NCT03738228	Phase I IB2/IIA with PAN, IIB/IIIB/IVA with Pelvic or PAN n = 40	Atezolizumab before and/or with standard CRT	Primary outcome: T-cell receptor beta clonal expansion Secondary outcomes: DLT, T-Cell Receptor clonality, PD-L1 expression
**(ATOMICC)** A Randomized, Open Label, Phase II Trial of Anti-PD1, TSR-042, as Maintenance Therapy for Patients with High-risk Locally Advanced Cervical Cancer After Chemo-radiation (34) NCT03833479	Phase II IB2, IIA2, IIB with pelvic nodes and IIIA, IIIB, IVA, or any stage with PAN, post standard CRT + cisplatin with curative intent n =132	Experimental anti-PD1 (TSR-042) as a maintenance therapy following standard CRT.	Primary outcome: PFS at 30 months Secondary Outcomes: Adverse Events, Overall Survival, Health related quality of life, fatigue, pain
**(NiCOL)** A Phase-I Study of Nivolumab in Association with Radiotherapy and Cisplatin in Locally Advanced Cervical Cancers Followed by Adjuvant Nivolumab for up to 6 Months (35) NCT03298893	Phase I IB2-IVA no PD-L1 expression required n = 21	Single arm concurrent nivolumab with CRT (IMRT+SIB, no brachytherapy) followed by 5 months of nivolumab alone	Primary Outcome: DLT Secondary Outcomes: ORR, PFS, circulating tumor DNA, Tumor microenvironment, PD-L1
**NCT02635360** Pembrolizumab and Chemoradiation Treatment for Advanced Cervical Cancer (36)	Phase II Stage IB2-IIA with + pelvic lymph nodes, Stage IIB-IVA any nodal status, IVB if metastases to PAN only n = 88	Pembrolizumab following standard CRT vs concurrently with standard CRT	Primary outcomes: immunologic effects in tumor and peripheral blood mononuclear cells Secondary Outcomes: HPV E2, E7, CD8+ T-cells, FoxP3+ T-regulatory Cells
**(ATEZOLACC)** Randomized Phase II Trial Assessing the Inhibitor of Programmed Cell Death Ligand 1 (PD-L1) Immune Checkpoint Atezolizumab in Locally Advanced Cervical Cancer (37) NCT03612791	Phase II Locally advanced Cervical Cancer n = 190	Atezolizumab concurrent then continued (max 20 weeks) with standard CRT vs standard CRT alone	Primary outcome: PFS up to 24 months
**NCT01711515** Chemoradiation Therapy and Ipilimumab in Treating Patients with Stages IB2-IIB or IIIB-IVA Cervical Cancer (38)	Phase I Stage IB2-IIA with PAN and IIB/IIIB/IVA with positive Lymph nodes n = 34	Sequential Adjuvant ipilimumab following concurrent weekly cisplatin and EFRT	Primary outcome: Maximum Tolerated Dose Secondary outcomes: DLT, ORR, HPV specific T-cell kinetics and HLA-subtypes

GOG, Gynecologic Oncology Group; EFRT, Extended Field Radiation Therapy; NRG (NSABP/RTOG/GOG), National Surgical Adjuvant Breast and Bowel Project/Radiation Therapy Oncology Group/Gynecologic Oncology Group; PD-L1, programmed death ligand 1; PAN, para-aortic nodes; CRT, chemoradiation; DLT, dose limiting toxicities; PD1, programmed death receptor-1; PFS, progression free survival; IMRT, intensity modulated radiation therapy; SIB, simultaneously integrated boost; ORR, overall response rate; DNA, Deoxyribonucleic acid; HPV, human papillomavirus; HLA, human leukocyte antigen.

An interesting phase II trial of pembrolizumab, NCT02635360 (**Table 2**), is exploring multiple immunological effects of both sequential and concurrent use of pembrolizumab in standard CRT+ brachytherapy (36). Measurements of HPV E2, E7 specific CD8+ T-cells, T-regulatory cells (T-regs), and Plasminogen activator inhibitor-1, a marker of immunosuppressive transforming growth factor-beta and rate of complete metabolic response on PET-CT imaging will be measured at 12 weeks post CRT. Safety, PFS, and OS will be followed to 5 years. The Nivolumab in Association with Radiotherapy and Cisplatin in Locally Advanced Cervical Cancers (NiCOL) trial (**Table 2**), a phase I study that aims to look at dose-limiting toxicity (DLT) of nivolumab as well as ORR and PFS when immunotherapy is continued 5 months post initial treatment with nivolumab + CRT (35). IMRT will be used to deliver pelvic radiotherapy (45 Gy) with simultaneous integrated nodal boost (54 Gy). Nivolumab will be given in a flat dose of 240 mg every 2 weeks or 1 mg/kg every 2 weeks. TME, circulating tumor deoxyribonucleic acid heterogeneity, and Tumor PD-L1 will be measured up to 2 years. The Anti-PD-1, TSR-042, as Maintenance Therapy for Patients with High-risk Locally Advanced Cervical Cancer After Chemo-radiation (ATOMICC) trial is a phase II trial using anti PD-1, TSR-042 as consolidation therapy post standard CRT (**Table 2**) (34). This trial hypothesizes an increased PFS by taking advantages of “the ideal microenvironment” created after radiation. A fixed 500 mg TSR-042 dose every 3 weeks for the first four doses followed by a fixed 1,000 mg dose every 6 weeks will be given for up to 24 months. PFS, OS and multiple quality of life measures will be followed to 30 months.

Atezolizumab is a humanized monoclonal antibody immune checkpoint inhibitor (ICI) that selectively binds to PD-L1 to stop the interaction between PD-1 and B7. The antibody still allows interaction between PD-L2 and PD-1. This antibody is being explored in locally advanced cervical cancer in a randomized phase II trial, the Assessing the Inhibitor PD-L1 Immune Checkpoint Atezolizumab in Locally Advanced Cervical Cancer (ATEZOLACC) trial (**Table 2**) (37). Patients must have bulky disease and/or positive nodes (both pelvic and para-aortic nodes (PAN) allowed). The primary objective is to evaluate clinical benefits of adding atezolizumab concurrently with standard CRT then continued as adjuvant for a total maximum 20 cycles. The primary outcome measure is PFS using RECIST (v1.1) or death up to 24 months. Ipilimumab as sequential adjuvant therapy to CRT is being explored in Phase I clinical trial NCT01711515 (**Table 2**) (38). The primary objectives are maximum tolerated dose and DLT following concurrent weekly cisplatin and EFRT in newly diagnosed lymph node positive cervical cancer. Eligible patients include stage IB2/IIA with PAN and stage IIB/IIIB/IVA with any positive lymph nodes (pelvic and/or PAN). Secondary objectives include PFS and evaluation of site of recurrence at 1 year along with chronic toxicities. HPV subtype specific T-cell kinetics, human leukocyte antigen immune markers and PET-CT changes after treatment will also be explored with follow-up to 1 year.

### Combination Anti-VEGF and Anti PD-1 Therapy

There are two ongoing clinical trials evaluating OS when combining anti-VEGF with anti PD-1 immunotherapy. The Efficacy and Safety of BCD-100 (Anti-PD-1) in Combination With Platinum-Based Chemotherapy with and without Bevacizumab as First-Line Treatment of Subjects with Advanced Cervical Cancer, (FERMATA) trial, is a phase III trial combining paclitaxel, platinum-based chemotherapy and an anti-PD-1 (BCD-100) with or without bevacizumab as first line therapy (40). The trial patients include histologically confirmed cervical SCC either progressive/recurrent (previously treated) or initial treatment of advanced stage (IVB) disease. The Platinum Chemotherapy Plus Paclitaxel with Bevacizumab and Atezolizumab in Metastatic carcinoma of the Cervix (BEATcc) Phase III trial is exploring the addition of azetolizumab to platinum chemotherapy, paclitaxel and bevacizumab in 404 patients with Stage IVB, persistent or recurrent cervical cancer (41). Both SCC and adenocarcinoma histology, as well as prior cisplatin-based CRT, will be balanced between the two arms. These trials are set to complete in 2024 and 2023 respectively. Any outcome differences from these combined therapies are anticipated to spur more multi targeted therapy trials.

### CTLA-4 Inhibition in Cervical Cancer

CTLA-4 is a cell marker constitutively expressed on T-reg cells that binds costimulatory molecule B7, thereby suppressing T-cell activity and the subsequent cytokine production required for a full immune response (20, 42). CTLA-4 was identified as a prognostic marker for cervical cancer, with a higher susceptibility in Asian populations, and studies have shown that low T-reg frequencies were associated with longer OS (43–45). Additionally, Qin et al. found that mutations in the CTLA-4 gene were positively associated with tumor mutation burden in cervical cancer (46). Mutational burden has been shown to correlate with and potentially predict response to immunotherapy (47). This would suggest exploration of CTLA-4 as a meaningful target in cervical cancer.

Concordantly, there are several studies by the Agenus corporation currently examining the role of CTLA-4 inhibitor, or AGEN1884, in cervical cancer. The first of these studies, NCT02694822 is a phase I/II trial assessing the safety, pharmacokinetics, and pharmacodynamics of AGEN1884 in patients with advanced solid cancers or cancers refractory to PD-1/PD-L1 inhibitors (48). This study was subsequently expanded to include cervical solid tumors, NCT03495882 (49). The final AGEN1884 trial, NCT03894215 is a randomized, non-comparative, phase II clinical study observing the efficacy and safety of AGEN2034, a PD-L1 inhibitor versus a placebo, and AGEN2034 + AGEN1884 in subjects with advanced cervical cancer after failed chemotherapy. As of April 2020, the combination of AGEN1884 and AGEN2034 has demonstrated an ORR of 26% in second-line cervical cancer treatment with a median follow-up of 12 months (50). Studies examining second generation CTLA-4 inhibitors are in development which are fragment crystallizable engineered to generate a response in a larger number of patients. Currently, the phase 1 trial using AGEN1181 ± AGEN2034 in advanced solid tumors is open to enrollment in advanced cancers (NCT03860272) (51).

### CTLA-4 Inhibitors and Radiation Therapy

**Figure 1:**
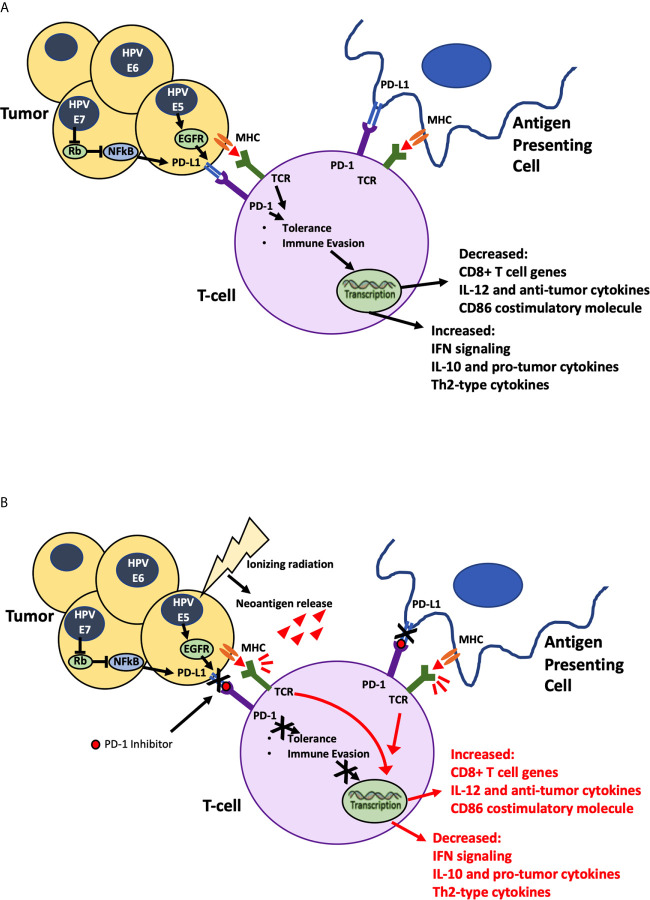
Ionizing Radiation in Combination with PD-1 inhibitor. **(A)** HPV mediated Oncogenic proteins E5, E6 and E7 hypothesized to cause increase in PD-L1 expression allowing tumor cells to evade identification by immune cells. **(B)** Ionizing radiation damages tumor cells causing neoantigen release, priming the immune system to attack, while PD-1 inhibitor blocks stimulation of immune evasion pathways. Combination of radiation and immunotherapy hypothesized to stimulate robust synergistic attack against tumor cells.

GOG 9929 is a phase I study exploring the use of ipilimumab sequentially after CRT for cervical cancer patients with International Federation of Gynecology and Obstetrics (FIGO) 2009 stages IB2/IIA with PAN and stage IIB/IIIB/IVA with pelvic or PAN (**Table 2**) (32). This high-risk group has a historically poor outcome with CRT alone (52). Lymph node metastasis in cervical cancer has been shown to have a 3-year cause specific survival (CSS) of 29% vs those without lymph node metastasis having CSS of 73% (32, 52, 53). GOG 9929 included concurrent weekly cisplatin, EFRT with nodal boost and intracavitary brachytherapy, followed by four treatments of ipilimumab. Included in GOG 9929 is tracking of immune biomarkers over the course of multimodality treatments. Immune responses including CD4+ and CD8+ T-cell activation *via* expression and activation of Inducible T-cell co-stimulator (ICOS) and PD-1, as well as HPV genotype specific E6/E7 oncogene specific responses were seen following initial CRT (32). These increases in lymphocyte activation appear to show CRT may have a “priming of the immune system” effect. Subsequent administration of ipilimumab sustained the activation of CD8+ T-cells and increased the activation of CD4+ T-cells above initial CRT levels (**Figure 1**). This revealed that in cervical cancer with high risk for recurrence and metastasis, ipilimumab may fortify the patient’s own antitumor response once activated by CRT. Preliminary results at the American Society of Clinical Oncology (ASCO) 2017 meeting were presented including 34 patients enrolled of which 19 patients were evaluable. At a median follow-up of 12 months in the patients who received ipilimumab, PFS was 81%, with OS reported as 90%. There were no major toxicities reported. There was suggestion of a significant correlation of increased PFS (p = 0.049), (**Table 2**) and OS (p = 0.036) for patients with increased activation of CD4+ cells expressing ICOS and PD-1 (32). While this is a possible association with increased immune activation and lower risk of progression and death, these results are preliminary and limited to 19 evaluable patients. Mature results as well as study with larger patient numbers are required to determine if immune-response can be utilized to tailor cervical cancer treatment with CRT.

## Special Considerations of Immunotherapy

As with all advances in oncology treatment, it is important to not only recognize the potential benefits of highly personalized cancer treatments and immunotherapy, but also the barriers to use and limitations. Cervical cancer presents an enormous burden to women in less developed countries, where the majority of cases present in socially disadvantaged women with advanced stage disease. In these settings there is limited or no access to immunotherapy or the necessary medical environment for implementation (54).

There is also the concern about durable response with the use of ICIs. As of 2018 a publication showed six ICIs had received approval for more than 10 cancer types (55). There are occasions when ICIs are used off-label for patients who have exhausted all other means of treatment, popularly known as “desperation oncology”. From 2011–2018 the estimation of the percentage of patients eligible for ICIs has shown a drastic increase from 1.54 to 43.63%. Concordantly, the estimated response to ICIs has increased over the years. With the approval of ipilimumab in 2011, patients with Non-Small Cell Lung Cancer (NSCLC) had an estimated response percentage of 0.14% (95% CI, 0.13–0.15%), which staggered until 2015. During this time nivolumab and pembrolizumab were introduced and the estimated response rate rose to 12.46% (95% CI, 12.37–12.54%) by 2018.

However, further analysis into patient eligibility and the efficacy of ICIs has raised some considerable concerns. Individually, the estimated eligibility and response to ICIs show a positive trend (55). In 2014 the ratio of response to indications peaked and eventually dropped as more ICIs were approved (56). This ultimately widened the gap between patients who are eligible for ICIs and actual benefit or response to the drugs. There is also concern for the under and over estimation of patient eligibility. ICIs are usually not approved as an early treatment option, therefore in settings such as GI cancers which have high mortality rates before later therapies can be used, ICI eligibility is severely miscalculated as it only accounts for a small subset of this population. On the other hand, in the setting of NSCLC, where a significant number of patients have long term survival with chemotherapy, the number of patients eligible for ICIs are underestimated as survivors are not considered in the eligibility criteria. Additionally, with the practice of desperation oncology, a standard does not exist to assess outcomes, which may further underestimate the number of patients affected by ICIs.

Finally, the use of ICIs has shown a correlation with hyperprogressive disease (HPD). There exist various definitions of HPD spanning from doubling of the tumor growth rate to increased tumor burden (57). While HPD is not exclusive to patients receiving ICIs, it occurs at a higher rate in patients who receive them and ultimately leads to poorer patient outcomes.

## Tumor Microenvironment

### Understanding the Effects of Radiotherapy and Chemotherapy on TME

With the promising potential of combining immunotherapy with chemotherapy and radiation, it is important to understand the effects these treatments have on the TME especially when considering concomitant or sequential treatments. Cisplatin has been shown to increase the recruitment of dendritic cells that promote CD8+ T-cells, and stimulate the type I interferon pathway, which ultimately improves host immunity against cancer cells (58). Radiation was shown to increase overall immune tumor response in mice when administered with immunogenic agents including vaccines and Toll like receptor agonists (59). Specifically, one study administered a tumor associated antigen vaccine to mice with carcinoembryonic antigen positive tumors who then received brachytherapy (60). The results interestingly showed that CD8+ T-cells of mice who received radiation coded for additional tumor antigens not included in the original vaccine. This appeared to define a pathway for the abscopal effect. A study by Nesslinger et al. evaluated the serum concentrations of patients with prostate cancer who received hormone therapy or radiation therapy after radical prostatectomy. Patients who received surgery alone did not generate an immune response, while the highest tumor antibody concentrations (in decreasing order) were for hormone therapy, brachytherapy and finally EBRT (61). Overall, these studies support that radiation has a synergistic immunological effect on the TME with measured tumor specific antigens.

There has been opposition to the therapeutic role of radiation on the immune system with suggestion the stimulatory and functional outcomes of the TME after radiation have yet to be carefully studied (62). There are also studies showing that radiation treatments can decrease the host’s immune response. Radiation was found to elicit undesirable immune changes such as decreased reactivity of T-cells to antigenic molecules, and increased expression of PD-L1 on CD4+ T-cells thought to decrease antigenic response (63). Moreover, lymphocyte counts in patients with invasive stage IB1 to IV cervical cancer were still found to be decreased in patients receiving EBRT ± cisplatin. In patients with HPV related cancer, radiation was found to create an adverse ratio of CD8+ T-cells:T-reg cells, in addition to increasing PD-L1 expression on CD4+ tumor cells. Overall, these findings suggest scenarios where radiation may be immunosuppressive and therefore possibly antagonistic to immunotherapy.

However, rather than try to omit radiation therapy, discussions should aim at finding optimal doses of radiation in combination with immunotherapy to yield synergistic effects. A study comparing standard four-field box and anteroposterior–posteroanterior techniques to bone marrow sparing intensity modulated radiation therapy (BMS-IMRT) found that BMS-IMRT can reduce the radiation dose to the lumbosacral spine bone marrow as well as decrease the volume of radiation to the pelvic bone marrow (64). These combined effects of bone marrow sparing constraints can decrease bone marrow suppression and other hematologic toxicities associated with radiation therapy (64, 65).

The synergistic role of radiation when administered with immunotherapy continues to be expounded. Multiple ongoing cervical cancer clinical trials using sequential or concurrent immunotherapy with CRT have included examination of immunological markers, some following changes throughout and beyond treatment (**Table 2**). Increased understanding of how the TME is altered by CRT and immunotherapy will help guide future combinations and timing of immunotherapies to hopefully foster the development of immune-response driven individualized therapy.

## Conclusions

While the radiotherapeutic management of cervical cancer has advanced with technological advancements, the inclusion of cisplatin-based concurrent chemotherapy has remained largely unchanged. There is significant need for improved outcomes in patients with locally advanced disease. Using anti-VEGF inhibitors to counter the upregulated angiogenesis from HPV-induced E5 oncoproteins in cervical cancer seems a logical consideration. Anti-VEGF therapy, combined with radiation and chemotherapy, has been shown to be effective in initial studies but requires randomized data to determine possible inclusion in standard of care. Immunotherapy targeting the PD-1/PD-L1 pathways has similarly shown promise in treatment of advanced cervical cancers. With increasing evidence of PD-L1 expression from cervical intraepithelial neoplasia to metastatic disease, immunotherapy (with or without additional systemic or local therapy) may potentially have a therapeutic role across several stages of cervical cancer. Preliminary results of CTLA-4 inhibitors in combination with CRT show the ability of radiation to act as an immune primer for further enhancement by immunotherapy. Multiple ongoing studies exploring the concurrent use of immunotherapy with standard of care CRT look to elucidate the importance of therapy timing in addition to provide further definition into the importance of immunological response. Future investigation into the optimal radiotherapy fractionation and sequencing are also required to fully understand the potential synergy of CRT targeted therapies. Of particular interest are studies investigating biomarkers that can potentially be utilized to tailor treatment strategies for individual patients according to tumor and immune response.

## Author Contributions

All authors contributed to the conception, design, and writing of the manuscript. All authors contributed to the article and approved the submitted version.

## Conflict of Interest

The authors declare that the research was conducted in the absence of any commercial or financial relationships that could be construed as a potential conflict of interest.
